# Family Growth and Survival Response to Two Simulated Water Temperature Environments in the Sea Urchin *Strongylocentrotus intermedius*

**DOI:** 10.3390/ijms17091356

**Published:** 2016-08-29

**Authors:** Yaqing Chang, Xiaofei Tian, Weijie Zhang, Fenjie Han, Shun Chen, Mi Zhou, Zhenguo Pang, Shoubing Qi, Wenping Feng

**Affiliations:** Key Laboratory of Mariculture & Stock Enhancement in North China’s Sea, Ministry of Agriculture, Dalian Ocean University, Dalian 116023, China; wangbei0303@yeah.net (X.T.); zhangweijie5217@163.com (W.Z.); fenjiehan@163.com (F.H.); CS18842608395@126.com (S.C.); 13342258953@163.com (M.Z.); pangzsr@gmail.com (Z.P.); qishoubing521@126.com (S.Q.); wpfeng90@163.com (W.F.)

**Keywords:** sea urchin, *Strongylocentrotus intermedius*, growth, survival, temperature, genotype-environment interaction

## Abstract

Heat tolerance is a target trait in the selective breeding of the sea urchin *Strongylocentrotus intermedius*, as it plays an important role in the survival and growth of cultured *S. intermedius* during summer. We investigated family growth and survival response to two temperature treatments to evaluate the genotype by temperature interaction (GEI) in the family selection of *S. intermedius*. Sea urchins from 11 families were exposed to two simulated water temperature environments—high temperature (HE) and control temperature (CE)—for 12 months, with each experiment divided into four periods (P1, stress-free period I; P2, stress-full high period; P3, stress-response period; and P4, stress-free period II) based on the temperature changes and the survival. Test diameter (TD), body weight (BW), and survival rate (SR) in HE and CE were measured monthly. Effects of family, temperature, and family-temperature interaction on TD, BW, SR, and specific growth rate (SGR) for BW were examined. In CE, BW differed significantly between families in P2, P3, and P4, while TD differed significantly between families in P3 and P4 (*p* < 0.05). In HE, family had significant effects on BW in P4, and on TD in P3 and P4, while temperature had significant effects on SR, TD, and BW in P3 and P4 (*p* < 0.05). GEI effects were not significant for TD or BW; however, family ranking changes revealed the existence of GEI in SR. The GEI results indicate the necessity of applying family selection in CE and HE for SR, but not for TD or BW. These results may provide a guide for aquaculture and selective breeding of *S. intermedius* under temperature pressure.

## 1. Introduction

The demand for sea urchins and their roe has exceeded supply in recent years. This has resulted in increasing interest in sea urchin aquaculture, especially for *Strongylocentrotus intermedius*, which is distributed along the coast of Hokkaido, Japan and the Russian Far East [1]. Owing to the high quality of roe of this species, it has become one of the most economically valuable sea urchins [2]. *Strongylocentrotus intermedius* was introduced to China from Japan in 1989 and artificial breeding has been continuous since then. Currently, *S. intermedius* is one of the major aquaculture species in China and the annual production of roe is considerable [3]. The growth of sea urchins is sensitive to temperature [4,5,6,7]. In *S. intermedius*, with regard to the specific growth rate (SGR), the significant effects of environmental factors, such as diet [8] and stocking density [9], have been documented. However, the effect of temperature on SGR is rarely seen. Temperature is one of the major environmental factors that affect gonad production and gonad quality in sea urchins [4,5,10]. The optimal temperature for *S. intermedius* in the Sea of Japan is below 20 °C. Water temperature higher than 25 °C can lead to a decrease in test diameter (TD), morbidity, and even death [11]. Previous studies have shown that gonad production of *S. intermedius* at 25 °C was much lower than at 10 °C [11], with temperatures above 21 °C decreasing gonad production [6]. In addition, high temperature can restrict larval development, as indicated by another study [12] reporting that the embryos and pluteus of *S. nudus* cannot live above 25 °C. From these studies, it is evidently clear that temperature is an important environmental factor that must be considered in culturing and breeding programs for sea urchins. However, the summer water temperature in the majority of China’s seas is greater than 25 °C, which has limited the aquaculture of *S. intermedius* with this species only cultured in the Liaoning and Shandong coastal areas. The most effective solution to this problem is to develop a new heat-resistant seed of the sea urchin.

Based on previous studies showing that the heritabilities of growth traits in *S. intermedius* are moderate to high [13,14], selective breeding programs for *S. intermedius* have been conducted in China since 2004. After four generations of selection, the growth traits and gonad quality of *S. intermedius* have been greatly improved. However, stress-resistance traits for *S. intermedius* are not selected as target traits in the breeding program. Selective breeding is an efficient method for achieving genetic improvement in aquatic species, especially for stress-resistance trait improvements. Selection of stress-resistance traits in aquatic species has typically focused on performance across different environments. A previous study compared BW growth differences between matrilineal full-sib families of *Paralichthys olivaceus* at high-temperature-stress (28 °C) and estimated the heritabilities as 0.15–0.38 [15]. In addition, their results suggested a chronic temperature lethal experiment for testing the heat-tolerance ability of *P. olivaceus.* Genetic parameters of shell length, shell width, BW, and resistance to low salinity for the families in *Haliotis diversicolor supertexta* have also been studied [16]. The trait of low-salinity resistance for each family was shown by calculating the survival rate (SR) of animals in low salinity at 16‰ for 48 h. No significant correlations between the major growth traits and SR were obtained. Therefore, combined breeding techniques are suggested for the selection of low-resistance strains in *H. diversicolor supertexta.* Genotype-environment interactions (GEI) are widespread in natural and culture environments [17] and occur when there are changes in the expression levels of genes regulating a trait change between environments that have selective pressures [18]. For breeding of specific species, a breeding plan for stress-resistance traits should be prepared based on the results of production performance in different environments and GEI to obtain genotypes that can adapt to different environments.

In the present study, two simulated water temperature environments were used to investigate GEI in *S. intermedius*. The aim of the study was to select high survival, fast-growing families under high temperature to expand the potential for aquaculture of the sea urchin in this region.

## 2. Results

### 2.1. Recorded Traits

In the present study, 220 sea urchins were equally distributed between 11 black plastic cages in two simulated water temperature environments. The temperature of HE environment ranged from 7.6 to 25.5 °C, while the temperature of CE environment ranged from 7.1–24.2 °C (Table 1). The SR of stress-response period in each family ranged between 25% and 85% in the high temperature (HE) treatment, and between 75% and 100% in the control temperature (CE) treatment (Table 2). The results of the analysis of variance (ANOVA) by general linear model (GLM) are shown in Table 3. Family and temperature had a significant effect on test diameter (TD) and BW (*p* < 0.05) in the stress-full high period, stress-response period, and stress-free period II (Table 3). However, the GEI (family × temperature) had no significant effect on these growth traits during any of the periods (Table 3).

### 2.2. Stress-Free Period I

**Figure 1:**
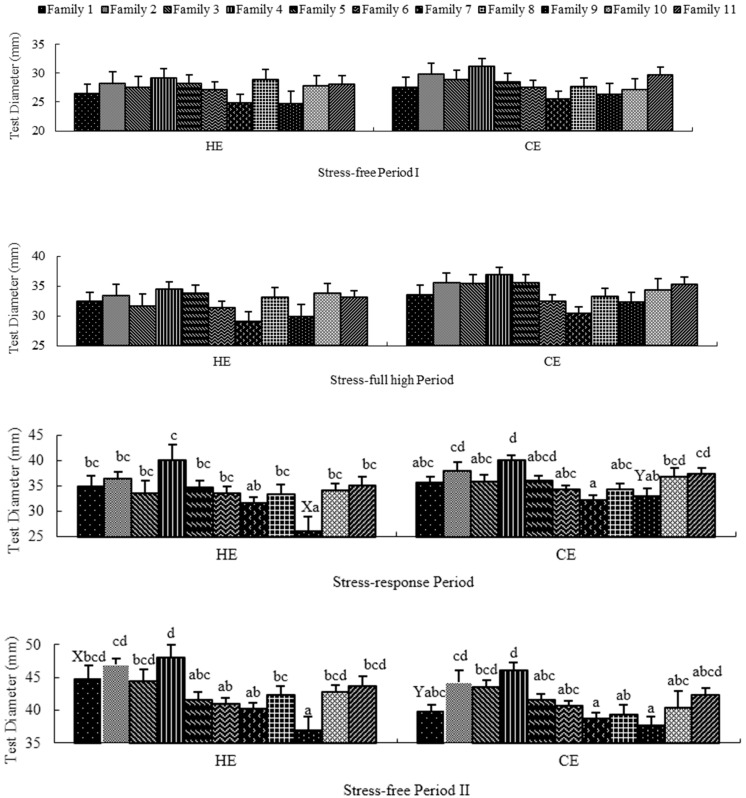
Ranking of families for test diameter in high temperature environment and control temperature environments. The histogram bar indicates mean and standard error of each separate family. In each environment, lower-case letters (a,b,c,d) indicate significant differences between families (*p* < 0.05), and upper-case letters (X,Y) indicate significant differences between environments (*p* < 0.05).

**Figure 2:**
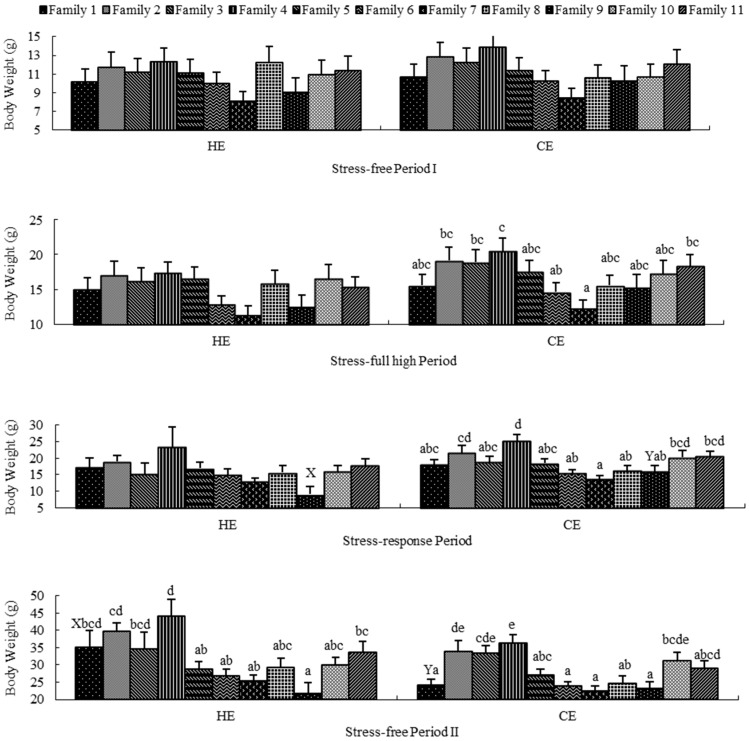
Ranking of families for body weight in high temperature and control temperature environments. The histogram bar indicates mean and standard error in each separate family. In each environment, lower-case letters (a,b,c,d,e) indicate significant differences between families (*p* < 0.05), and upper-case letters (X,Y) indicate significant differences between environments (*p* < 0.05).

Family had no significant effect on growth traits (TD and BW) in HE and CE (Figure 1 and Figure 2) during P1. In addition, temperature had no significant effect on growth traits in each family. Moreover, the GEI (family × temperature) had no significant effect on growth traits (Table 3). For the variance components of variables, the proportion of variance explained by family were low for TD and BW (1.564% and 1.782%, respectively). The proportion of variance explained by temperature for TD and BW were 0.137% and zero, respectively. The proportion of variance explained by GEI for both growth traits were zero. There was no mortality in either of the simulated environments during this period.

### 2.3. Stress-Full High Period

Family had no significant effect on growth traits in HE and on TD in CE, but did have a significant effect on BW in CE (*p* = 0.033) (Figure 1 and Figure 2) during P2. In addition, temperature and GEI had no significant effects on growth traits in each family (Figure 1 and Figure 2, Table 3). For the variance components of variables, the proportion of variance explained by family for TD and BW were 3.746% and 5.309%. The proportion of variance explained by temperature for TD and BW were 2.534% and 1.827%. The proportion of variance explained by GEI for both growth traits were zero. The SR of all families in each simulated environment was 100% during this period.

### 2.4. Stress-Response Period

Family did have a significant effect on TD in HE (*p* < 0.05) and on both TD (*p* = 0.001) and BW (*p* < 0.001) in CE (Figure 1 and Figure 2) during P3. Temperature had no significant effect on growth traits in each family with the exception of family 9 (*p* < 0.05) (Figure 1 and Figure 2). The GEI had no significant effect on growth traits (Table 3). The proportion of variance explained by family for TD and BW were 12.465% and 14.570%. The proportion of variance explained by temperature for TD and BW were 4.770% and 4.696%. The proportion of variance explained by GEI for both growth traits were zero.

Table 2 shows differences of SR between families and between environments tested by Chi-square tests. Both family and environment significantly affected the SR of the sea urchin (*p* < 0.05). Table 2 also shows the family ranking of SR in the two simulated temperature environments. The multiple inversions of family ranking between the two environments reveal the existence of GEI. For instance, family 7 had the highest SR in HE, but had the second lowest SR in CE. On the other hand, there were families (e.g., family 5 and family 1) with small-scale re-rankings, and even no re-rankings.

**Figure 3:**
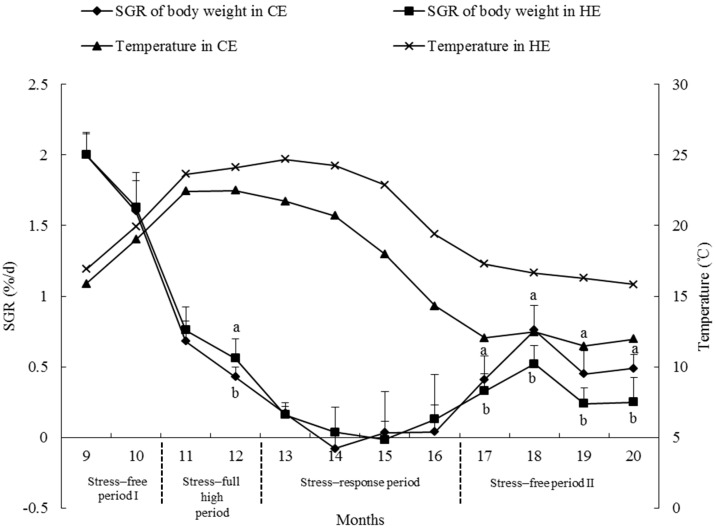
Comparison of specific growth rate of body weight between high temperature (HE) and control temperature (CE) environments. In each month, lower-case letters (a, b) indicate the difference between temperatures, different letters represent significant difference (*p* < 0.05).

In HE, the BW specific growth rate (SGR) of the surviving sea urchin was close to zero (Figure 3), signifying that these surviving sea urchins did not grow. To determine the relationship between body size and survival, BW between surviving and deceased sea urchins (15.14 ± 7.02 g and 15.17 ± 8.16 g, respectively) were compared using one-way ANOVA. No significant differences were discovered between them (*p* = 0.984).

### 2.5. Stress-Free Period II

Family had a highly significant effect on the growth traits in each temperature treatment (*p* ≤ 0.001) (Figure 1 and Figure 2) during P4. However, temperature had no significant effect on growth traits in each family, with the exception of family 1 (*p* < 0.05). Moreover, the GEI had no significant effect on growth traits (Table 3). The proportion of variance explained by family for TD and BW were 15.463% and 20.686%. The proportion of variance explained by temperature for TD and BW were 2.883% and 3.943%. The proportion of variance explained by GEI for both growth traits were zero. The SR in this period was the same as in P3, except for family 8 (45%) and family 11 (45%) in HE, and family 8 (85%) in CE.

### 2.6. Growth Rate

The rate of growth is measured as the SGR of BW. Figure 3 shows the SGR of BW in all sea urchins measured monthly in the two simulated water temperature environments (HE and CE). The results reveal that the SGR of BW decreased with time and increasing temperatures. When the temperature reached 23–25 °C and sustained for three months, the SGR of BW became zero, and sometimes negative. As the seasons changed, the seawater temperature decreased and the SGR of BW began to increase again, but did not reach the original rapid growth. Sea urchins in the CE environment had a significantly higher SGR of BW than those in the HE environment at the age of 12 months in the stress-full high period (P2) (*p* < 0.05). During stress-response period (P3), temperature differences between HE and CE did not significantly affect the SGR of BW. In stress-free period II (P4), sea urchins in HE environment had significant higher SGR of BW than those in CE environment (*p* < 0.05).

## 3. Discussion

### 3.1. Family Effect

Family had a significant effect on growth traits (TD and BW) in *S. intermedius*, but this effect was only displayed gradually and the impact on growth slowly increased during the 12-month experimental period. Family did not significantly affect the growth traits during P1, but did significantly affect growth traits during the other three periods (results by GLM analysis). This differed from previous studies [19], where it was reported that family had a significant effect on the growth of *S. intermedius* during early growth stages (six months old to 10 months old). One potential reason for this difference is that sea urchins selected by Zhang et al. [19] were similar in size whereas. in the present study. the sea urchins were chosen randomly, which increased the variance within families. Other aquatic species have been studied that are significantly affected as a consequence of family, e.g., rainbow trout *Oncorhynchus mykiss* [20,21] and silver-lip pearl oyster *Pinctada maxima* [22]. The proportion of variance explained by family for TD and BW gradually increased from the P1 to P4 (1.564%–15.463% in TD and 1.782%–20.686% in BW, respectively), which means that the family variance may account for only a small portion of phenotypic variance in the early growth stage of *S. intermedius*, but a large proportion of phenotypic variance after P2. At harvest, the proportion of variance explained by family for BW had reached 20.686%. Zhang et al. estimated the proportion of variance explained by family for TD and BW as 0.465%–0.944% [19]. These results were much lower than our results. The reason might be the proportion of variance explained by error in our study was less than that in the study of Zhang et al. [19]. The two environments in this study differed in temperature only. While the environments in the previous study differed in temperature, tank, illumination, stocking density, and so on. The single factor in this study might decrease the proportion of variance explained by error so that relatively increase the proportion of variance explained by family. The proportion of variance explained by family of sea urchin in this study was higher than that obtained in the Pacific oysters, *Crassostrea gigas* [23], because the family effect differs between species.

### 3.2. Temperature Effect

The seasonal range of environmental temperature of *S. intermedius* is from −5–25 °C in the Sea of Japan [24] and 1–25 °C in the Yellow Sea near Dalian [11]. Thus, the temperature environment of CE (ranging from 5.6–24.2 °C) is a typical environment for culturing *S. intermedius.* It should be noted that the control temperature environment CE was the temperature of seawater pumped from the Yellow Sea near Dalian (38.87° N, 121.56° E) without heating or cooling. We changed the water every three days, so the water temperature would be affected by room temperature and not be identical with the sea water. When sea urchins were placed in the HE treatment, they had to tolerate a new environment with a temperature that was significantly different from the temperature in CE. Nonetheless, these significant temperature effects on growth traits were shown only in one family during P3 and another during P4. Moreover, the proportion of variance explained by temperature was only 0%–4.770%, which is lower than that obtained in other aquatic species [23,25,26]. This might be because the seasonal changes in sea water temperature were sequential. Thus, the small changes in temperature in the present study were still in the acceptable range of several of the families and, therefore, would not have a significant impact on growth. This result shows the feasibility of introducing *S. intermedius* to seas of low latitudes.

In the present study, a significant temperature effect on SR was detected in the majority families, except in family 7. High temperature led to death of the experimental sea urchins. When water temperature reached 24 °C, deaths began to occur. Although the water temperature then began to decrease gradually, deaths of sea urchins continued for a further four months. It is supposed that deaths in this study might be the consequence of accumulated temperature, which destroyed the normal physiological function of the sea urchins. Thus, we assume that long-term observation is required in order to ascertain survival accurately. The results confirm the study of Chang et al. [11] who observed that high temperature (>25 °C) can lead to massive deaths in *S. intermedius*. Additionally, Tajima et al. [27] detected no deaths of *S. intermedius* at 20 °C but 100% mortality at 25 °C. No significant differences in BW were found between surviving and deceased sea urchins, suggesting that there was no correlation between size and heat resistance under temperature pressure in the present study. In the red sea urchin *Mesocentrotus franciscanus*, one study revealed that large individuals are more likely to survive than small ones at high temperature [28],which is different to the results obtained from the present study. One possible reason for this difference is that in the previous study [29], 2714 sea urchins from 18 sites in the USA were examined, whereas in the present study only 220 sea urchins in the HE treatment were tested. Further study should be conducted in this field.

### 3.3. Genotype-Environment Interaction

For growth traits, no interaction was identified between genotype and temperature with the ranking of the families being similar for both temperature environments and during each temperature period (P1–P4). These results suggest that *S. intermedius* families selected for good growth performance in HE are likely to grow faster in CE, which means that when growth traits are chosen as the target trait, temperature would not affect the selection result. Our findings agree with the results of several fish studies that showed either no interaction or only a weak interaction between two different environments [29,30,31]. However, contrary results have been reported for small *S. intermedius* [19], in which three laboratory environments that commonly used in breeding programs for *S. intermedius* were assessed. Highly significant GEI effects were recorded [19]. A possible reason for this difference in the GEI is different environments. Water temperature was the unique experiment factor in this study, while the environment factor in the previous study [19] was a compound factor, including temperature, density, tank, and other factors.

The SR of families ranked differently between HE and CE. This revealed the existence of GEI. From the family ranking of SR, family can be separated into two groups: the changing type, wherein the SR ranking of the family changed between HE and CE, and the stable type, wherein the SR ranking remained the same between the two temperature treatments. Although the changing type had multiple inversions of family ranking, some families (families 7 and 10) survived well at high temperatures and could be selected as parents for the next generation. The stable type (families 1 and 5) had similar family rankings and a high SR in both temperature environments, meaning they could be the families selected to produce the next generation with high temperature tolerance. Therefore, the GEI in SR should not be overlooked in breeding programs for *S. intermedius* with the present study increasing our understanding of GEI in SR and providing guidance for breeding strategies of *S. intermedius.*

### 3.4. Growth Rate

High temperature in HE treatment significantly suppressed the SGR of BW of sea urchins at the age of 12 months during P2, suggesting that the temperature 22.47 °C (average temperature in CE during P2) might be close to a fringe where the growth of the sea urchin begins to be suppressed by high temperature. This temperature is comparable to previous studies by Chang et al. who concluded that *S. intermedius* of middle size had a slower test diameter growth at 22 °C than at 19 °C [11]. Lawrence et al. also reported that food consumption of *S. intermedius* were significantly affected at 22 °C [6]. Although the temperature in CE during P3 decreased and was significantly lower than that in HE, the SGR of BW did not differ significantly between the two temperatures. The reason might be that sea urchins in both HE and CE would consume a majority of energy in tolerating high temperatures, or high temperatures in both HE and CE would bring some physiological damage to the sea urchins. The sea urchins in HE had significantly higher SGR of BW than those in CE during P4. That was because the temperature in HE was significantly higher than in CE. More importantly, the temperature in HE during P4 had decreased to an average temperature 16.66 °C. Chang et al. [11] concluded that adult *S. intermedius* had the largest test diameter growth rate at 16 °C. Our results with regard to the SGR of BW suggested that, the water temperature lower than 22.5 °C in summer and maintaining at 16.7 °C in winter would raise the growth rate of *S. intermedius*. In addition, age was negatively correlated with the SGR of BW. *Strongylocentrotus intermedius* is a cold-water species that occurs naturally in cold-water environments. In the early growth stage, water temperature is usually suitable for growth, with the majority of energy intake of *S. intermedius* used for body growth. During the reproductive season, the majority of energy will be used for reproductive effort and growth will gradually decrease [32,33,34,35].

## 4. Materials and Methods

### 4.1. Experimental Animals

Family lines of *S. intermedius* were maintained at the Key Laboratory of Mariculture and Stock Enhancement in North China’s Sea, Ministry of Agriculture, Dalian Ocean University, China. Two batches of F_1_ generation families containing 293 full-sib families were separately constructed in 2006 and 2007. In 2009, using the second batch of F_1_ as parents, the second batch of F_2_ generation containing 39 full-sib families were constructed [16]. Based on the second batch of F_2_ generation that had been produced, 27 families with the highest mean BW values were selected (13 July 2011). After that, the five sea urchins with the highest BW were selected from each selected family. Every selected sea urchin was induced to spawn by injection with KCl solution (0.5 mol/L, 1–2 mL/ind); the sperm of 36 male parents and eggs of 72 female parents were collected. One sire was randomly mated to two dams (inbreeding was avoided). Seventy two full-sib families were eventually produced. The hatching and rearing methods were almost the same as the description by the previous study [1]. When the families were eight months old (13 March 2012), 40 individuals were randomly chosen from 11 randomly chosen families as experimental animals. The BW was 3.60 ± 2.90 g (mean ± SD).

### 4.2. Experimental Design

The high temperature environment, HE, was selected based on the higher temperature of the northern East China’s Sea (30.14° N, 122.51° E). The control temperature environment, CE, was the temperature of seawater pumped from the Yellow Sea near Dalian (38.87° N, 121.56° E) without heating or cooling. Hydrological data revealed that the temperature in the northern East China Sea is higher than that of the northern Yellow Sea, especially during summer. After being cultured at Dalian since 1989, *S. intermedius* has adapted to the temperature environment of the northern Yellow Sea. The temperature of CE (ranged from 5.6 to 24.2 °C) was that of ambient sea water. The temperature of HE was 1–5 °C higher than ambient temperature. Two in-door recirculating seawater systems, each having one ~1 m^3^ polypropylene tank (170 cm length, 90 cm width, 70 cm depth) were utilized to achieve the target temperature. Water flow of each recirculating seawater system was ~100 L·min^−1^. Excluding the water temperature, all other environmental conditions in the two treatments remained the same.

Forty sea urchins from each of the eleven families were separated into two groups. One group containing 20 individuals of each family was placed in each black plastic cages (18 cm side, 60 cm depth). Eleven cages were randomly placed into a recirculating seawater system for either the HE or CE treatment. After placement in the cages, the sea urchins were maintained for two weeks without food.

Subsequently, all cages were provided with excess food (the alga *Laminaria japonica*) to ensure ad libitum feeding of sea urchins during the experiment. The seawater (31‰–32‰) was strongly aerated and exchanged every three days in both tanks. The seawater temperature was recorded three times per day in each tank (Table 1). TD and BW were measured monthly using digital calipers (measuring accurate to 0.01 mm) and an electronic balance (measuring accurate to 0.01 g), respectively (as per the protocol of Zhang et al. [36]) from 13 March 2012 to 13 March 2013. The SR in both treatments in each family was recorded monthly, and the BW of each dead individual in each family was recorded in the HE treatment. Based on temperature changes and the survival, the growth period was separated into four different periods: P1, stress-free period I, in which both the temperatures of HE and CE were lower than 20 °C; P2, stress-full high period, in which both the average temperatures of HE and CE were higher than 20 °C, both the survival rates were 100%; P3, stress-response period, in which the experimental sea urchins began to die; and P4, stress-free period II, in which both the average temperatures of HE and CE were lower than 20 °C, the experimental sea urchins basically stopped dying. 

The SGR of BW was calculated using the formula:

*SGR =* 100 × (ln *X*_f_*−* ln *X*_i_)/*T*

where, *X*_f_ is the final BW, *X*_i_ is the initial BW, and *T* is the number of days.

### 4.3. Statistical Analysis

A generalized linear models procedure was used to evaluate the data. The effects of family, temperature, and family-temperature interaction (genotype-environment interaction, GEI) on TD and BW were examined using the following model:

*Y*_ijk_ = μ + family_i_ + temperature_j_ + (family × temperature)_ij_ + *e*_ijk_,

where *Y*_ijk_ is the phenotypic observations of each individual, μ is the general mean, family_i_ is the family effect, temperature_j_ is the temperature effect, (family × temperature)_ij_ is the GEI, and *e*_ijk_ is the residual error.

In order to examine the family re-ranking effects between environments and detect the temperature effect on growth traits in each family, the family effect was re-examined in each temperature environment, and the temperature effect was re-examined in each family using one-way ANOVA. Duncan’s multiple comparisons were made between families. Variance components of family, temperature, and family-temperature interaction were evaluated using the restricted maximum likelihood method. Temperature differences between periods were examined by one-way ANOVA and Duncan’s multiple comparisons. The differences of BW between living and dead individuals were tested in the HE treatment using one-way ANOVA. Chi-square test was used to analyze the significance of SR between environments and between families. The temperature effect on the SGR of BW in each month was examined using one-way ANOVA with families as replicates. All analyses were conducted in SPSS version 16.0 (SPSS, Inc., Chicago, IL, USA). The significance level of all analyses was set as *p* < 0.05.

## 5. Conclusions

In summary, the present study shows that the family effect exerts a gradually increasing role in the growth of *S. intermedius*. Temperature has a significant effect on survival and growth rate (the SGR of BW). The results show no GEI effect for growth, but an effect on survival. Breeders could enhance selective efficiency under temperature pressure by looking more closely at survival. The present study provides guidance for aquaculture and breeding programs of *S. intermedius*, particularly under temperature pressure.

## Figures and Tables

**Table 1:** Environmental conditions of the two simulated temperature environments.

	Temperature (°C)								pH *	Salinity (‰)	Illumination (lx)
	Stress-Free Period I		Stress-Full High Period		Stress-Response Period		Stress-Free Period II				
	8–10 Months Old		11–12 Months Old		13–16 Months Old		17–20 Months Old				
	Mean ± SD	Range	Mean ± SD	Range	Mean ± SD	Range	Mean ± SD	Range	Range	Range	Range
HE	17.15 ± 3.46 ^Xa^	7.60–23.20	23.94 ± 0.42 ^Xb^	22.20–25.30	22.88 ± 2.46 ^Xc^	17.60–25.50	16.66 ± 0.71 ^Xa^	14.20–19.30	7.70–8.02	31.85–32.75	0–490
CE	16.13 ± 3.52 ^Xb^	7.10–21.30	22.47 ± 0.52 ^Yc^	20.90–24.20	18.78 ± 3.14 ^Yd^	8.60–23.90	12.06 ± 1.47 ^Ya^	5.60–14.90	7.89–8.14	31.98–32.80	

For each temperature in each period, lower-case letters (^a,b,c,d^) indicate the difference between periods, different letters represent significant difference (*p* < 0.05). Upper-case letters (^X,Y^) indicate the difference between HE and CE, different letters represent significant difference (*p* < 0.05). * pH, salinity and illumination are the ranges for the entire experiment (8–20 months old).

**Table 2:** Ranking of survival rate of families (from high to low survival) after high temperature in two simulated environments. Differences between families and between temperature environments were tested by Chi-square test.

HE		CE	
Family	Survival Rate	Family	Survival Rate
7	85% ^Xd^	11	100% ^Ye^
5	70% ^Xc^	2	95% ^Yd^
10	55% ^Xb^	5	95% ^Yd^
1	50% ^Xb^	1	90% ^Yc^
8	50% ^Xb^	6	90% ^Yc^
9	50% ^Xb^	8	90% ^Yc^
11	50% ^Xb^	9	90% ^Yc^
2	45% ^Xab^	10	90% ^Yc^
6	35% ^Xa^	4	85% ^Yb^
3	30% ^Xa^	7	85% ^Xb^
4	25% ^Xa^	3	75% ^Ya^

Lower-case letters (^a,b,c,d,e^) indicate the difference of survival rate between families, different letters represent significant difference (*p* < 0.05); Upper-case letters (^X,Y^) indicate the difference between simulated temperature environments, different letters represent significant difference (*p* < 0.05); no letter or the same letter means no significant difference (*p* > 0.05).

**Table 3:** Effects of family, temperature, and genotype-environment interaction on growth traits and their variance component proportions in the four periods. Effects of family, temperature, and genotype-environment interaction on growth traits were tested by general linear models and their variance component proportions were tested by restricted maximum likelihood method.

	Stress-Free Period I		Stress-Full High Period		Stress-Response Period		Stress-Free Period II	
**Test Diameter (mm)**								
	***p***	Var % *	***p***	**Var %**	***p***	**Var %**	***p***	**Var %**
Family	0.102	1.564	0.005	3.746	<0.001	12.465	<0.001	15.463
Temperature	0.257	0.137	0.009	2.534	0.014	4.770	0.027	2.883
Family × temperature	0.997	0.000	0.994	0.000	0.767	0.000	0.873	0.000
Error	–	98.299	–	93.720	–	82.765	–	81.654
**Body Weight (g)**								
	***p***	**Var %**	***p***	**Var %**	***p***	**Var %**	***p***	**Var %**
Family	0.080	1.782	0.001	5.309	<0.001	14.570	<0.001	20.686
Temperature	0.468	0.000	0.022	1.827	0.010	4.696	0.003	3.943
Family × temperature	0.997	0.000	0.992	0.000	0.867	0.000	0.426	0.000
Error	–	98.218	–	92.864	–	80.734	–	75.371

* Var % represents the proportions of total phenotypic variation explained by each variable.
